# The Arabidopsis GPI-Anchored LTPg5 Encoded by *At3g22600* Has a Role in Resistance against a Diverse Range of Pathogens

**DOI:** 10.3390/ijms21051774

**Published:** 2020-03-05

**Authors:** Muhammad Amjad Ali, Amjad Abbas, Farrukh Azeem, Mahpara Shahzadi, Holger Bohlmann

**Affiliations:** 1 Department of Plant Pathology, University of Agriculture, Faisalabad 38040, Pakistan; amjad.abbas@uaf.edu.pk (A.A.); mahpara015@gmail.com (M.S.); 2 Centre of Agricultural Biochemistry and Biotechnology, University of Agriculture, Faisalabad 38040, Pakistan; 3 Institute of Plant Protection, Department of Crop Sciences, University of Natural Resources and Life Sciences, 1180 Vienna, Austria; 4 Plant Biotechnology Lab, Department of Bioinformatics and Biotechnology, GC University, Faisalabad 38040, Pakistan; farrukh@gcuf.edu.pk; 5 Grassland Economics and Systems Analysis Laboratory, College of Pastoral Agriculture Science and Technology, Lanzhou University, Gansu 730000, China

**Keywords:** lipid transfer protein, antimicrobial peptide, Arabidopsis, *Heterodera schachtii*, plant defense

## Abstract

Arabidopsis contains 34 genes for glycosylphosphatidylinositol (GPI)-anchored LTPg proteins. A motif analysis has placed these into four groups. With one exception, all are produced with a signal peptide and are most likely attached to the cell membrane via the GPI anchor. Several of the LTPg genes across the four groups are downregulated in syncytia induced by the beet cyst nematode *Heterodera schachtii*. We have here studied *At3g22600* encoding LTPg5, which is the most strongly downregulated LTPg gene. It is mainly expressed in roots, and a promoter::GUS line was used to confirm the downregulation in syncytia and also showed downregulation in galls of the root knot nematode *Meloidogyne incognita*. In contrast, infection with bacteria (*Pseudomonas syringae*) and fungi (*Botrytis cinerea*) led to the induction of the gene in leaves. This diverse regulation of *LTPg5* indicated a role in resistance, which we confirmed with overexpression lines and a T-DNA mutant. The overexpression lines were more resistant to both nematode species and to *P. syringae* and *B. cinerea*, while a knock-out mutant was more susceptible to *H. schachtii* and *P. syringae*. Thus, LTPg5 encoded by *At3g22600* is part of the Arabidopsis resistance mechanism against pathogens. LTPg5 has probably no direct antimicrobial activity but could perhaps act by associating with a receptor-like kinase, leading to the induction of defense genes such as *PR1*.

## 1. Introduction

Plants are under constant pressure from various pathogens and pests. For protection, they produce physical barriers that can also be reinforced in response to pathogen attack. Furthermore, the defense system against these threats comprises constitutive and inducible production of various compounds, including small molecular weight secondary metabolites, proteins, and peptides. Antimicrobial peptides are not specific to plants as they are also produced by animals, fungi, and even bacteria. The major plant-specific antimicrobial peptide families are plant defensins, thionins, and lipid transfer proteins (LTPs) [1,2].

LTPs, as the name suggests, were originally described as intracellular transporters of lipids but have also been shown to have antimicrobial activity in vitro. LTPs usually contain eight cysteine residues which form disulfide bridges that stabilize the 3D structure of the protein which contains four alpha helices. However, LTPs are generally produced as preproteins with an N-terminal signal peptide for secretion into the apoplast. They are generally basic and contain a hydrophobic cavity involved in binding lipids or other non-polar substances. The 3D structure has been determined for several LTPs with different methods (reviewed by [3]). Several LTPs have been isolated from plants and have been shown to possess antimicrobial activity in vitro. These include, for instance, LTPs from radish [4], onions [5], barley and maize [6], Arabidopsis and Spinach [6], and *Coffea canephora* [7]. The latter was shown to have antimicrobial activity in vitro against several phytopathogenic fungi by increasing membrane permeability and by inducing the endogenous production of reactive oxygen. Arabidopsis DIR1 (*At5g48485*) on the other hand was found as a component for the signaling to the establishment of systemic acquired resistance [8]. LTPs have also been found to be important for resistance against abiotic stress. The *Nicotiana tabacum* NtLTP4 is, for instance, involved in salt and drought stress tolerance [9].

Originally, LTPs were divided into group 1 LTPs with approximately 90 amino acids and group 2 LTPs with approximately 70 amino acids [10]. According to Edstam et al. [11], 10 types are now recognized, with type G (LTPg) having a C-terminal sequence motif for the post-translational addition of a glycosylphosphatidylinositol (GPI)-anchor. The remaining LTPs without a GPI-anchor have been further divided into nine types based mainly on intron position and spacing of the cysteine residues.

Arabidopsis contains 34 LTPg genes [12]. They have been found to be involved in various developmental processes as well as stress responses. Several LTPgs are also involved in the development of pollen and seeds [13]. LTPg15 is, for instance, involved in the export of suberin monomers to the seed coat [14]. Loss of function mutants of LTPg1, 2, 5, and 6 have decreased resistance against the non-host powdery mildew fungus *Blumeria graminis* f. sp. *hordei* [15]. In case of LTPg1, the resistance mechanism seems to involve incorporation of the LTP into papillae, cell wall reinforcements produced by the plant at attempted penetration sites. However, the authors did not find a correlation between the composition of cuticular wax with the observed penetration phenotype of the *ltpg* mutants, indicating a possible direct antimicrobial activity of the studied LTPgs.

Plant pathogenic nematodes are a threat to agriculture worldwide. Some of the most devastating species are cyst nematodes. They invade the roots of their host plants as juveniles where they induce the development of a syncytium starting from an initial syncytial cell. The syncytium grows to a size of several hundred cells by incorporating neighbouring cells by partial cell wall dissolution. Cells within the syncytium are metabolically highly active, the large central vacuoles are fragmented into many small ones and nuclei are enlarged due to the endo-reduplication of DNA. All these changes are initiated by effectors produced by the nematode and injected into the host cells with their stylet. The syncytium is the only source of nutrients for the cyst nematodes throughout their life. Male nematodes leave their syncytium after a while to mate with females. Females stay at their syncytium and produce several hundreds of eggs that are contained in their body which grows to a lemon shape. The female body finally hardens and dies, thus becoming a cyst that can protect the eggs for many years until juveniles hatch under favourable conditions [16].

We have recently analyzed the transcriptome of syncytia induced by the beet cyst nematode *Heterodera schachtii* in Arabidopsis roots. We found that *LTPg5* (*At3g22600*) was one of the most strongly downregulated genes in syncytia, indicating that the LTPg5 protein might have an adverse effect on the development of syncytia or the nematode itself [17]. Here, we have studied the role of this gene and found that it plays a role in the Arabidopsis defense against a variety of pathogens including *Pseudomonas syringae*, grey mold *Botrytis cinerea* and root-knot nematode *Meloidogyne incognita* in addition to *Heterodera schachtii*.

## 2. Results

### 2.1. In Silico Characterization of LTPg Genes and Proteins Based on Different Attributes

The following attributes of LTPg genes and proteins were studied using different tools: amino acid length, protein molecular weight, isoelectric point (pI), the start and end point of the gene on chromosome, gDNA length, CDS (coding sequence) length, signal peptide cleavage site and the grand average of hydropathy (GRAVY). The protein length ranged from 116 (LTPg17) to 799 (LTPg32), with an average of 195 AA (amino acids). All LTPgs are produced as precursors with signal peptides, with the exception of LTPg6, indicating secretion of the mature proteins. Ten LTPgs showed highly acidic pI with LTPg25 showing the minimum value (4.05) and six LTPg proteins exhibited a pI above 8 with LTPg15 showing the maximum value of 8.66. LTPg5 is also highly acidic with a pI of 4.8. However, the part corresponding to the LTP domain has a pI of 8.22, while the extension has a pI of 4.11. GRAVY was positive for all the LTPg proteins except three, i.e., LTPg6, LTPg22, and LTPg32. The GPI anchor is attached at the ω-site at the C-terminus (Table S1).

### 2.2. Localization of Arabidopsis LTPg Genes on Chromosomes

**Figure 1:**
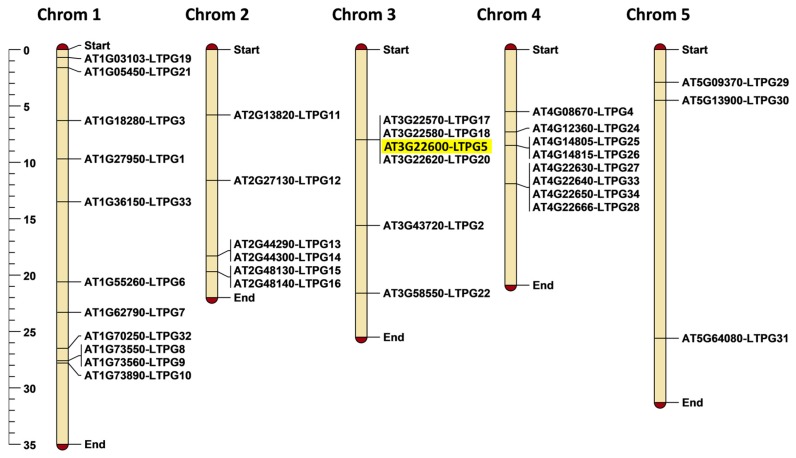
Chromosomal positioning of 34 LTPg genes on Arabidopsis chromosomes. The scale on the left side of the figure shows chromosomal length in mega base pairs (Mbp).

We used MapChart for mapping of all 34 *LTPg* genes on the Arabidopsis chromosomes. The results demonstrate that various chromosomes have a different number of LTPg genes (Figure 1). Chromosome 1 has the maximum number of 10 LTPg genes. Chromosomes 2 and 3 contain the same number of six genes each, whereas chromosome 4 and 5 contain seven and three LTPg genes, respectively. The localization of Arabidopsis LTPg genes on chromosomes displays that the *LTPg5* and three other genes *LTPg17*, *LTPg18,* and *LTPg20* are located together and form a cluster at chromosome 3.

### 2.3. Phylogeny, Gene Structure, and Conserved Motifs Analyses of LTPg Genes

**Figure 2:**
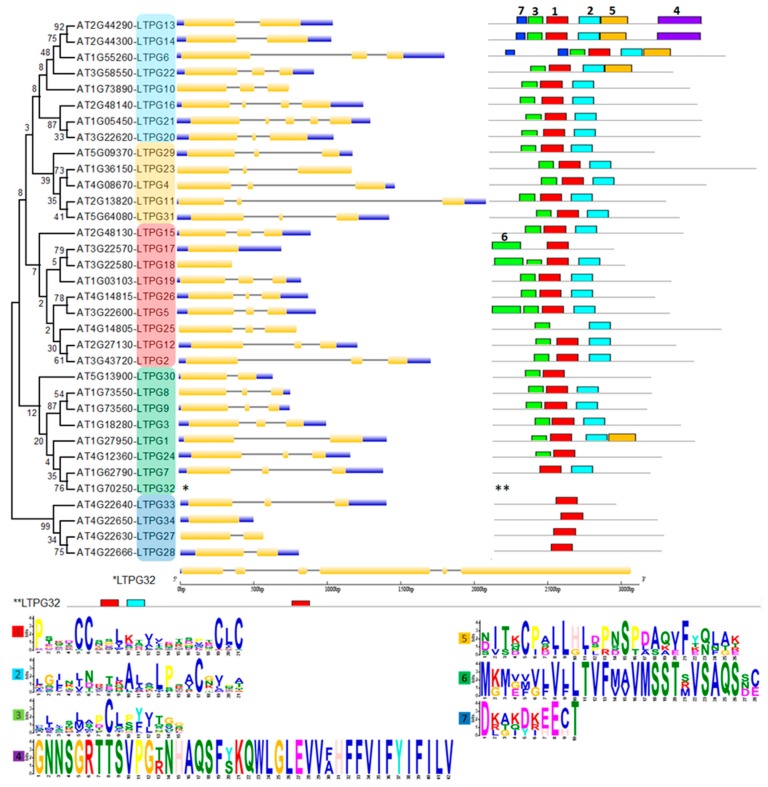
Phylogenetic tree, gene structure, and conserved motif analyses of LTPgs. Phylogenetic tree was developed using MEGA 7 software with alignment of the sequences through Clustal W followed by tree construction through maximum likelihood method based on the Whelan and Goldman (WAG) model. Intron-exon structure and motif analyses for each LTPg gene is given in front of the corresponding LTPg gene in the phylogenetic tree. Golden, blue, and thin grey colored lines show positions of exons, untranslated regions (UTRs) and introns, respectively. Scale below the figure compares the length of gene. Motif numbers assigned by MEME suite tool are mentioned on each motif. Motif heights depicting the significance of the match as taller are more significantly conserved than lower motifs. The sequence logos of all motifs are provided at the bottom. Gene structure and motif analysis of *LTPg32* is given below the tree due to large size of protein. Group 1 marked light blue and yellow, group 2 red, group 3 green, group 4 dark blue.

The phylogenetic tree of 34 LTPg proteins was made by using the maximum likelihood method based on the Whelan and Goldman (WAG) model. We combined phylogenetic relationship, gene structure analysis, and conserved motif analysis to study the similarities and differences among LTPg genes simultaneously (Figure 2).

**Figure 3:**
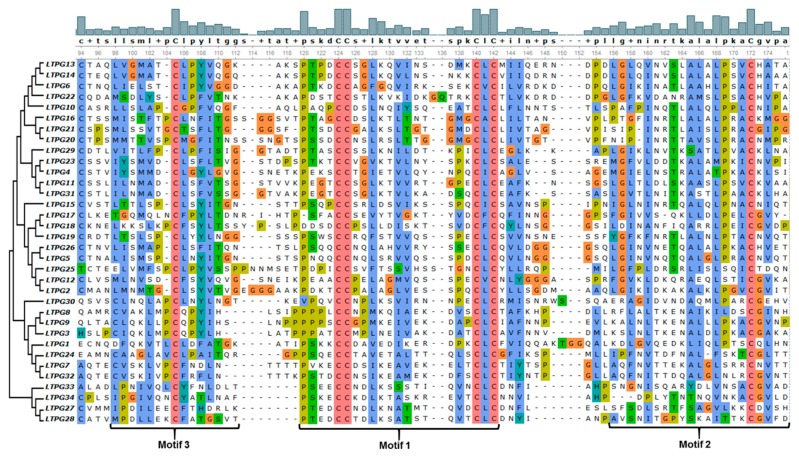
Multiple sequence alignment of the most conserved region of the LTPg proteins comprising the first three motifs.

The conserved motifs analysis through MEME Suite identified a total of seven different motifs, with motif 1, 2, and 3 found in the majority of the 34 LTPg proteins occurring 34, 27, and 27 times, respectively. These motifs correspond to the LTP domain containing six of the eight cysteine residues. Motif 1 was present in all the LTPg proteins except LTPg25 while being present twice in LTPg32. The sequence logos of different motifs clearly displayed a conservation of cysteine residues among the first three motifs (Figure 2). Cysteine was also highly conserved in motif 5 but to a lesser extent in motif 6 and 7. LTPg5 protein contained eight cysteine resides, which is an attribute of antimicrobial peptides. Multiple sequence alignment of the most conserved region of the LTPg proteins (motifs 3, 1, and 2) showed a high extent of sequence similarly (Figure 3).

The phylogenetic tree grouped LTPg proteins into four clusters. Group 1 was the biggest group that comprised 14 proteins and was divided into two subgroups with 9 and 5 proteins in the first and second subgroup, respectively. Most of the group members demonstrated a similar pattern of three exons and two introns in the gene structure with a few exceptions. For instance, *LTPg13* (*At2g44290*) and *LTPg14* (*At2g44300*) are tandem duplicates with only two exons and a single intron, while *LTPg16* and *LTPg21* display four and five exons with three and four introns, respectively. Substantial similarity was observed in the proteins of group 1 in terms of conserved motifs. All the proteins in this group possessed motif 1, 2, and 3. In addition, LTPg13, LTPg14, LTPg6, and LTPg22 had motif 5, while motif 7 was only characteristic of LTPg13, LTPg14, and LTPg6, where LTPg6 contained motif 7 twice. LTPg13 and LTPg14 are the only LTPgs that have motif 4 containing a large number of aromatic residues at the C-terminus.

**Figure 4:**
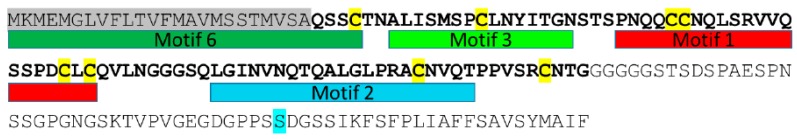
Sequence of LTPg5 protein, including the identified motifs. Signal peptide is shown in grey and the sequence corresponding to the nsLTP is in bold. Cysteine residues are marked yellow. Motifs are shown below the sequence. The GPI anchor is attached at amino acid 146 (S, marked blue).

Group 2 comprised of LTPg proteins that possessed highly conserved patterns of motifs and exon-introns in the gene sequence. All the members of this group had three exons and two introns with the exception of *LTPg17* and *18*, which are lacking introns. With a few exceptions, the members of this group contained the first three motifs. LTPg17 lacked motif 1 and 3. Motif 1, which is present in all other 33 LTPgs, was also missing in LTPg25 protein. Additionally, in this group, LTPg17, LTPg18, and LTPg5 displayed motif 6, containing the signal peptide and the first cysteine residue of the mature protein, which was not present anywhere else among the LTPg proteins. The motifs and the corresponding sequence of LTPg5 are also shown in Figure 4.

Eight proteins were grouped in cluster 3, where gene structure and motifs were highly conserved among the members. LTPg32 from this group is the longest gene among LTPgs and contained six exons and five introns. In addition, LTPg32 showed a duplication of motif 1, which is the main characteristic of all LTPgs except LTPg25. In this group, LTPg30 and LTPg24 lack motif 2, while LTPg7 and LTPg32 missed motif 3.

Group 4 comprised of four members with *LTPg27* and *LTP28* with one intron, *LTPg33* with two introns, while *LTPg34* lacked introns. However, all the members of this group only contained motif 1, which was highly conserved.

### 2.4. Expression Analysis of LTPg5

**Figure 5:**
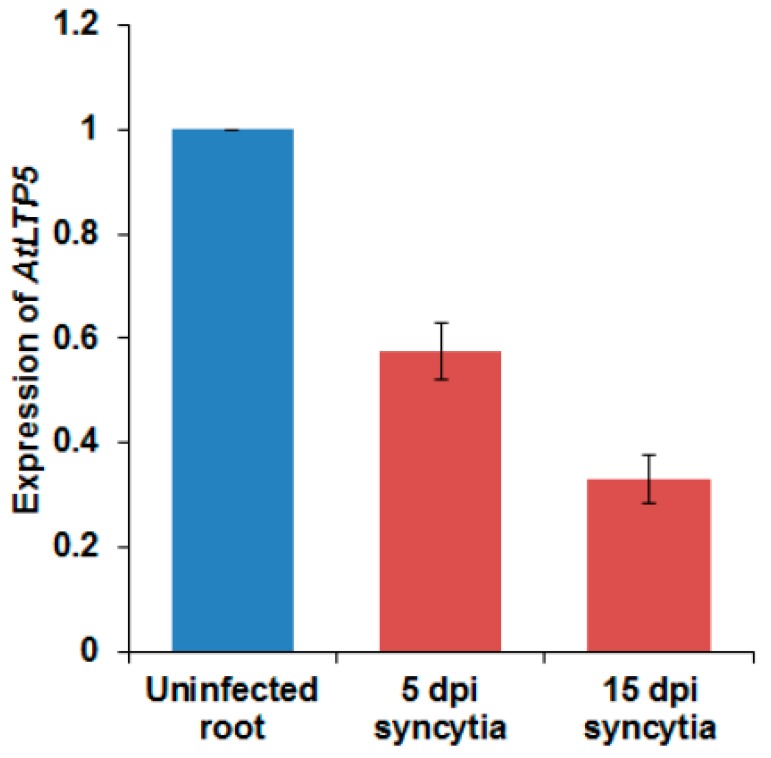
Expression of *LTPg5* in response to nematode infection in 5 and 15 dpi syncytia using quantitative real time PCR (qRT-PCR). The data included three independent biological replicates. The values are means ± SE, *n* = 3.

A previous transcriptome analysis of syncytia induced by the beet cyst nematode *Heterodera schachtii* in Arabidopsis roots [17] found that out of 34 GPI-anchor LTP coding genes, 12 genes showed significantly differential expression in syncytia (Table S2). Only two genes from this group (*At5g64080 = LTPg31* and *At2g27130* = *LTPg12*) were significantly induced in syncytia. However, 10 were significantly suppressed, including *LTPg5*, while 14 genes were not differentially regulated and eight genes were not present on the GeneChip. We found that the *LTPg5* gene was the most downregulated gene among all the *LTPg* genes (Table S2). We confirmed this with quantitative real time PCR (qRT-PCR) using syncytia cut out from infected roots five and 15 days after infection (dpi). In both samples, the expression was significantly downregulated, especially in the 15 dpi sample (Figure 5).

**Figure 6:**
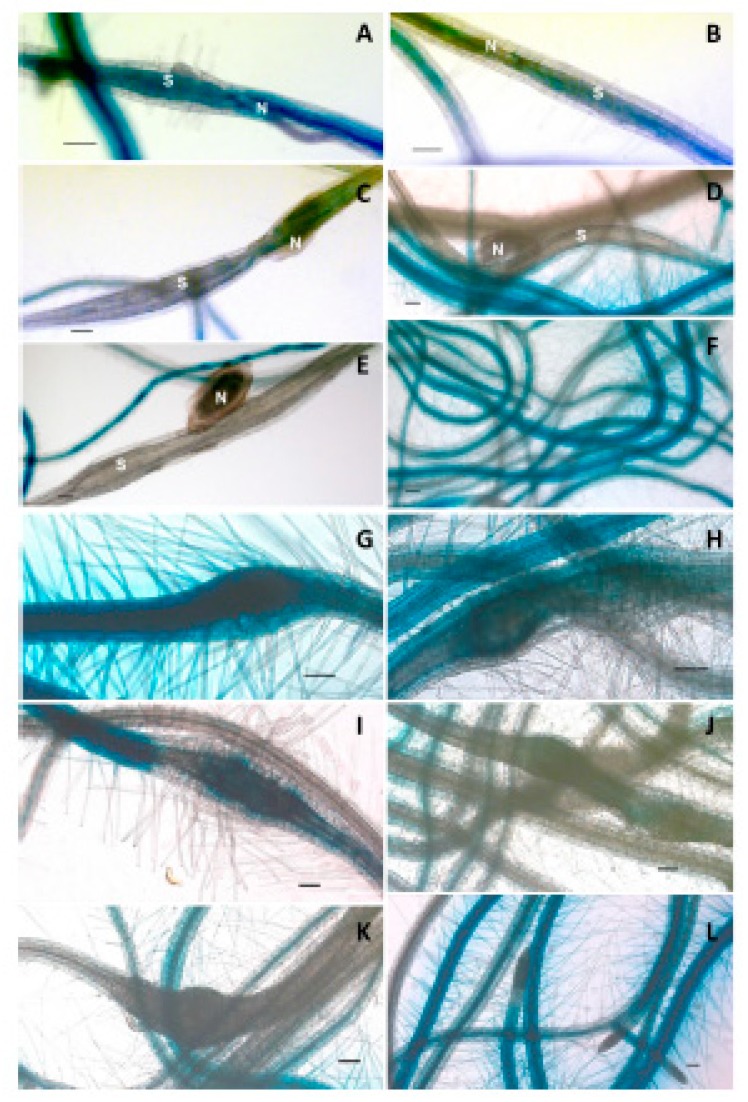
Promoter::GUS analysis of *LTPg5* in syncytia and galls induced by *H. schachtii* and *M. incognita,* respectively, at different time points. (**A**–**F**) represent GUS staining in 3, 5, 10, 12, and 15 dpi syncytia and uninfected roots, respectively, where N = nematode and S = syncytia. Similarly, (**G**–**L**) represent GUS staining in 3, 5, 10, 12, and 15 dpi galls and uninfected roots, respectively. Scale bar = 100 µm.

To further study the expression of *LTPg5*, we generated a promoter::GUS line. The inoculation of this line with *H. schachtii* confirmed the downregulation of the gene in syncytia (Figure 6A–F) starting at 5 dpi (Figure 6B), and onward up to 15 dpi (Figure 6C–F). The expression of the promoter::GUS construct was also seen in galls induced by *Meloidogyne incognita* (Figure 6G–I), which was first visible at 5 dpi. In older galls, expression was reduced as compared to the control uninfected roots, and absolutely no expression was observed in the 15 dpi galls.

**Figure 7:**
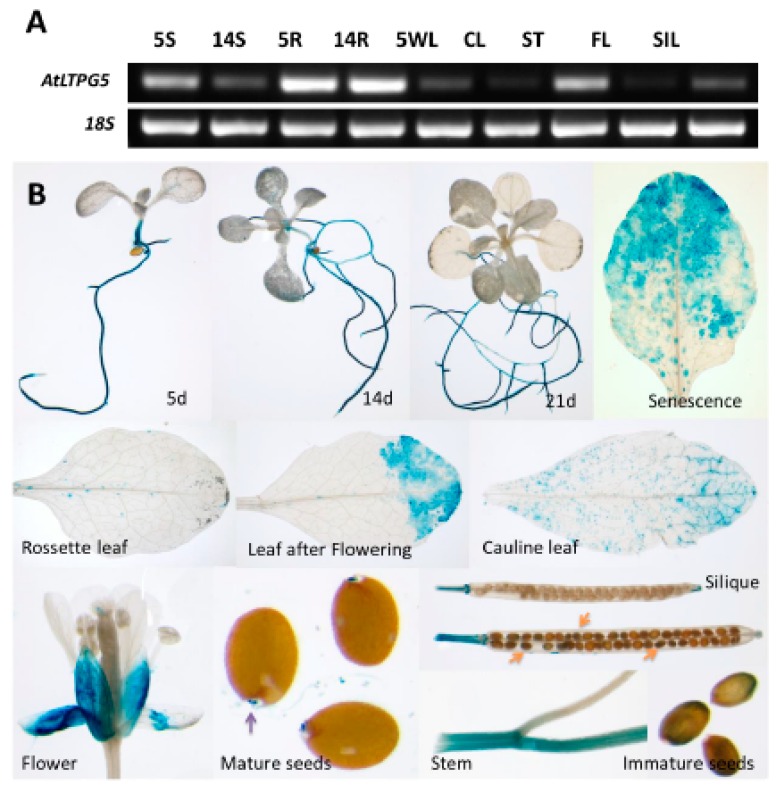
Expression analysis of different plant part and growth stages. (**A**) Reverse transcriptase PCR (RT-PCR) for the developmental regulation of *LTPg5.* RT-PCR using RNA isolated from seedlings grown on MS-medium (5S, 5-d-old shoots; 14S, 14-d-old shoots; 5R, 5-d-old roots; 14R, 14-d-old roots) or from plants grown on soil (5WL, 5-w-old leaves; CL, cauline leaves; ST, stems; FL, flowers; SIL, siliques). Primers for the 18S gene were used for control reactions. (**B**) *LTPg5* promoter activity in plant parts determined with GUS fusion. In mature seeds, micropylar endosperm gave strong staining.

We also studied the expression of this gene in different plant parts using reverse transcriptase PCR (RT-PCR) (Figure 7A) and further confirmed the expression using this GUS line for the expression in different plant parts (Figure 7B). Both these analyses displayed similar findings. The GUS expression was found in roots of seedlings, grown under sterile conditions on MS medium, at different ages (Figure 5) but not in cotyledons and young leaves of seedlings. In the rosette leaves of soil grown plants, expression was only found in some small spots while stronger expression was observed in leaves after flowering and in senescent leaves. Expression was also found in stems and cauline leaves. In flowers, there was expression only in sepals. Low expression was also found in seeds.

### 2.5. Role of AtLTPg5 in Resistance

**Figure 8:**
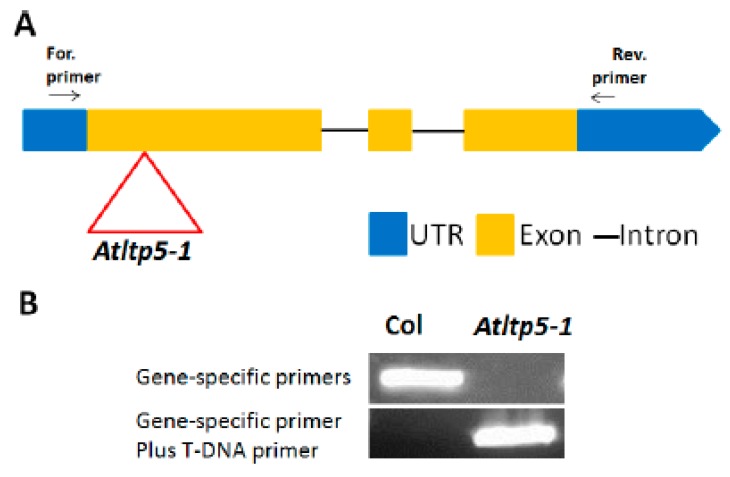
Knock-out mutant for *LTPg5*, (**A**) The T-DNA insertion (inverted triangle) in *ltp5-1* (SAIL_329_H03) (**B**) PCR with DNA from homozygous mutant and Columbia (Col).

**Figure 9:**
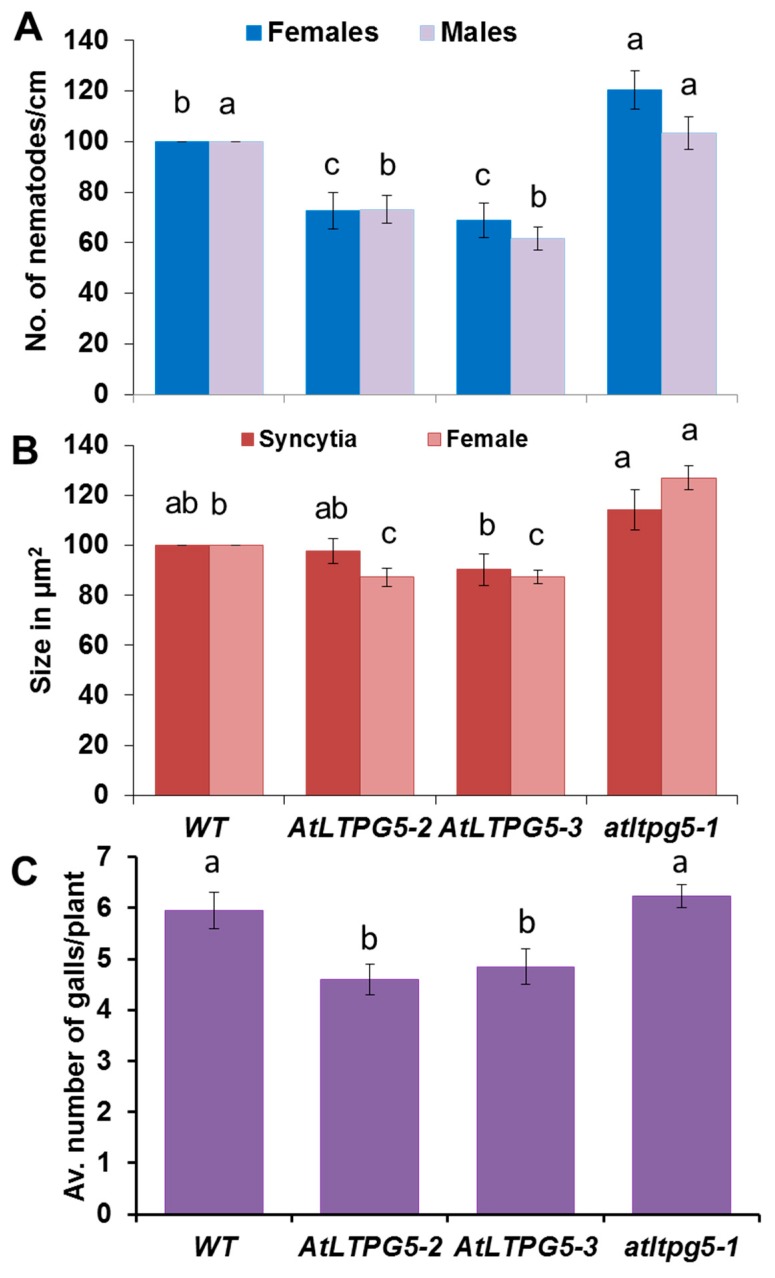
Nematode resistance test. The resistance of overexpression lines and a knock-out mutant of *LTPg5* was compared to wild type plants after infection with nematodes. (**A**,**B**) Infection with *H. schachtii*, (**A**) The number of male and female nemaodes per cm of root length calculated at 15 dpi setting the wild type as 100%. The infection rate is shown in column sets with different letters indicating significant differences (*p* < 0.05; ANOVA and LSD). The statistical significance was determined by three independent replicates for female and male nematodes separately. The values are means ± SE, *n* = 15. The bar shows standard error for the mean. (**B**) The size of female syncytia and female nematodes at 14 dpi. Eight syncytia were selected randomly from three independent replicates (total = 24), and the size of syncytia and associated female nematodes were determined. (**C**) *M. incognita* resistance test. The resistance of two overexpression lines of *LTPg5* was compared to wild type plants after infection with *M. incognita*. The number of galls per roots were counted at 15 dpi. The number of galls given in column sets with different letters indicate significant differences (*p* < 0.05; ANOVA and LSD). The statistical significance was determined by three independent replicates. Values are means ± SE, *n* = 12.

Having established that the *LTPg5* gene was especially expressed in roots and downregulated in nematode infection sites, we produced overexpression lines to study the effect of the encoded LTP protein on nematode infection. We included a mutant line (SAIL_329_H03) with insertion in the 1^st^ exon (Figure 8). The infection of these lines with *H. schachtii* is shown in Figure 9A,B. Both overexpression lines supported lower numbers of female and male nematodes than the wild type. The mutant, on the other hand, was more susceptible, supporting a significantly higher number of female nematodes than the wild type, while the number of male nematodes was not different from the wild type. We also measured the size of syncytia and of female nematodes. There was a significant difference between the wild type and one overexpression line (22–3) concerning the size of syncytia. The mutant syncytia were larger but the difference to syncytia in wild type roots was not significant. The size of female nematodes was significantly smaller on both overexpression lines and significantly larger in case of females developing on roots of the mutant. The effect of *LTPg5* on *M. incognita* is shown in Figure 9C. On both overexpression lines a significantly lower number of galls developed as compared to the wild type while there was no difference between the wild type and the mutant. Thus, overexpression rendered the plants less susceptible to both nematode species, while the mutant had only an effect on the size of *H. schachtii* females.

**Figure 10:**
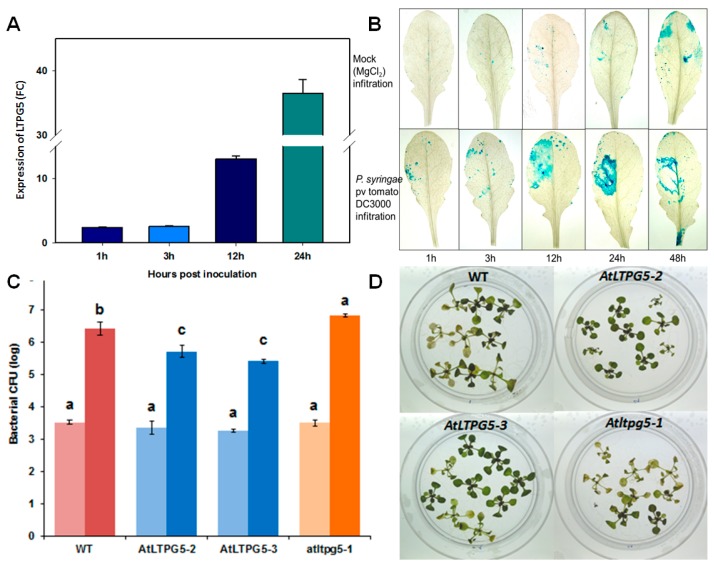
Expression and functional analysis of *LTPg5* in response to *P. syringae* pv tomato DC3000. (**A**) The expression of *LTPg5* in wild type plants in response to *P. syringae* pv tomato DC3000 was determined by qRT-PCR. The data included three independent biological and three technical replicates. The values are means ± SE, *n* = 3. The bar shows the standard error for the mean. (**B**) The GUS staining of rosette leaves of a promoter *LTPg5:*:GUS line after mock infiltration (MgCl_2_) and infiltration with *P. syringae* pv tomato DC3000 at different time points. (**C**) The overexpression lines, mutant, and wild type control were infected in plates by flooding according to [18]. The data were analysed for significance difference using ANOVA (*p* < 0.05) and LSD. The values are means ± SE. The light bars show mean CFU (colony forming units) at 1 hpi (hours after infection), while dark bars show mean CFU 72 hpi. (**D**) the overexpression lines showed less chlorosis and necrosis at 3 dpi as compared to Columbia wild type and mutant.

It has been reported that LTP genes are involved in resistance against other pathogens than nematodes. We have therefore tested if the *LTPg5* gene might also have an effect on other pathogens. The inoculation of Arabidopsis wild type plants with *Pseudomonas syringae* pv *tomato* DC3000 resulted in strong upregulation of the *LTPg5* gene after 12 h (Figure 10A). We confirmed this upregulation with the promoter::GUS line. The infiltration of the bacteria resulted in GUS expression around the infiltration site, which was strongest after 24 h (Figure 10B). This upregulation is supported by data from Genevestigator (Figure S1). The upregulation of the *LTPg5* gene in response to infection indicated that it might play a role in resistance against phytopathogenic bacteria. We therefore tested both overexpression lines and the mutant with *P. syringae*. Both overexpression lines were more resistant, having approximately 10-fold less bacteria at 3 dpi, while the mutant was significantly more susceptible with approximately 5 times more bacteria (Figure 10C). The difference was also visible in the phenotype of the plants where the overexpression lines showed less chlorosis and necrosis than wild type, while the mutant was more affected by the bacteria as compared to wild type (Figure 10D).

**Figure 11:**
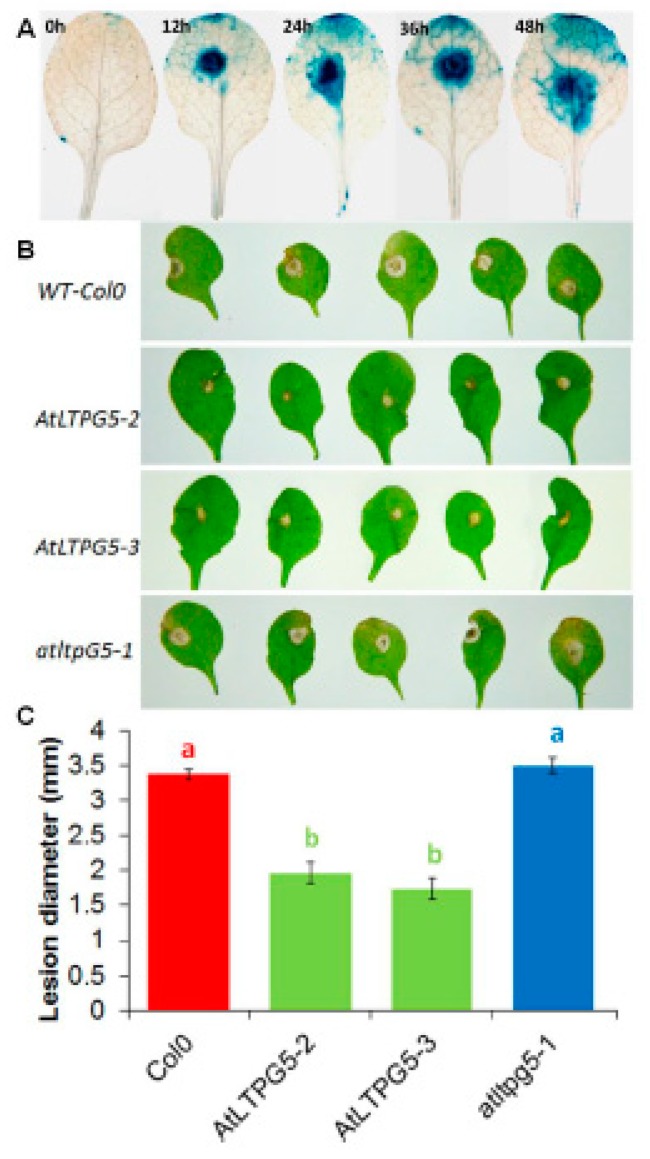
Expression and functional analysis of *LTPg5* in response to *B. cinerea* infection. (**A**) Expression analysis of promoter *LTPg5*::GUS line for GUS activity in response to *B. cinerea* at different time points. (**B**,**C**) *B. cinerea* infection test to evaluate the involvement of *LTPg5* in plant defense. The overexpression lines and mutant of *LTPg5* were compared with the wild type plant after infection. Plants were infected with *B. cinerea* by putting a 5-μL drop on the surface of 4-week-old leaves. Plants were grown in short day conditions with 16 h dark and 8 h light at 24 °C. (**B**) The representative pictures are shown at 5 dpi. (**C**) Lesion diameter in mm was determined at 5 dpi. For each line in a replicate, three plants were infected. Mean data from three experimental replications was subjected to analysis of variance and differences among mean determined by LSD at a 5 % significance level and *n* = 15. The values are means ± SE.

The effect on plant pathogenic fungi was tested with *Botrytis cinerea* as a model fungus. As with *P. syringae*, GUS expression was induced after inoculation of the leaves with *B. cinerea* spores already after 12 h (Figure 11A). This upregulation is supported by data from Genevestigator (Figure S1). The lesions that developed after infection with *B. cinerea* spores on the leaves of both overexpression lines were significantly smaller than on the wild type leaves (Figure 11B,C). There was no difference between the wild type and the mutant.

**Figure 12:**
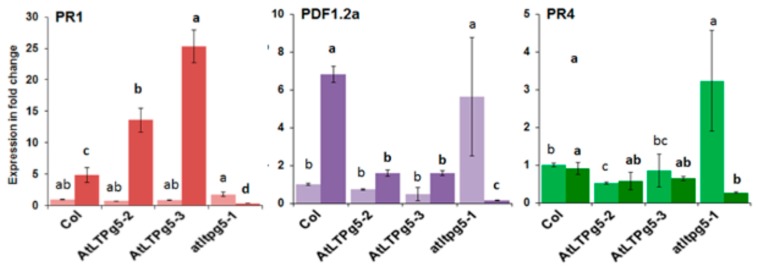
Expression of PR genes in response to *P. syringae* pv tomato DC3000 in *LTPg5* overexpression lines and mutant. The RNA was extracted from inoculated two-week-old seedlings on plates. The seedlings without root were harvested at 0 hpi and 24 hpi for three biological replicates (*n* = 3) for RNA isolation and qPCR. The light bars show expression in different genes at 0 hpi, while the dark bars display the expression at 24 hpi. The data were analysed for significance difference using ANOVA (*p* < 0.05) and LSD for 0 hpi and 24 hpi separately. The values are means ± SE. Different letters indicate significant differences for the expression of genes among the lines at different timepoints.

The resistance of the overexpression lines that we found could be due to a direct antimicrobial effect of the LTP or, considering that the LTP DIR1 is involved in systemic acquired resistance, might lead to the expression of defense-related genes. We therefore tested the expression of PR (pathogenicity related) genes after infection with *P. syingae* (Figure 12). We found a much stronger expression of the *PR1* gene in the overexpression lines, while expression was blocked in the mutant. A different picture was found for *Pdf1.2a* and *PR4*. Expression was relaxed in the mutant, being much stronger in non-infected plants but repressed after infection. For *Pdf1.2a*, the expression in the overexpression lines was lower after infection and for *PR4* there was no difference between wild type and overexpression lines.

In conclusion, our results show that the expression of *LTPg5* was induced by plant pathogenic fungi and bacteria, while it was downregulated in the feeding sites of plant pathogenic nematodes. In line with these results, the overexpression of the gene led to increased resistance in the transgenic plants. Thus, *LTPg5* is part of the plant resistance against pathogens.

## 3. Discussion

### 3.1. Arabidopsis LTPg Proteins

Arabidopsis contains 64 LTP genes, of which 34 code for LTPg proteins that are attached to the plasma membrane via a GPI anchor [12]. Our analysis has placed these proteins into four groups according to a motif analysis which identified a total of seven different motifs present in these proteins. There is a strong conservation of motifs 1, 2, and 3, which together contain six of the eight cysteine residues and are found in the majority of LTPg proteins and make up the typical LTP domain found also in the non-anchored LTPs. The size difference of the LTPg proteins is mainly due to the C-terminal extensions, which also contain the ω-site directing the attachment of the GPI anchor. Specific functions have only been described for some Arabidopsis LTPg proteins and may also depend on specific C-terminal extensions.

Several of the LTPg coding genes are tandem duplicates. The *LTPg5* gene, which is the main topic of this publication, is, for instance, part of a cluster of four genes on chromosome 3, which also contains *At3g22570* (LTPg17)*, At3g22580* (LTPg18), and *At3g22620* (LTPg20). While LTPg20 encoded by *At3g22620* belongs to Group 1, the other proteins from this cluster all belong to Group 3. While *LTPg5* and *At3g22620* are both downregulated in syncytia, they have a different function, as we will describe later.

### 3.2. LTPg Family Genes Are Downregulated in Syncytia

Of the 34 Arabidopsis LTPg genes, only two were significantly upregulated in syncytia induced by *H. schachtii*, while 10 genes were significantly downregulated [17]. The gene *LTPg5* was most strongly downregulated, indicating that the LTPg5 protein might be detrimental to nematode development. The downregulation of *LTPg5* was confirmed by the qRT-PCR of syncytia cut out from infected roots and a promoter::GUS line. The infection of the promoter::GUS line with *M. incognita* also found a downregulation of the gene in galls induced by this nematode in the roots. Thus, this gene is downregulated in feeding sites induced by different nematode species. The downregulation in syncytia is due to a DNA-binding protein GLAND4 produced by *H. schachtii* and the related species *H. glycnes* and injected via its stylet into syncytia. It binds to the promoter region of *LTPg5* and, in addition, it does so about 1 kb upstream of the transcription start site of *At3g22620* [19]. It can be proposed that a DNA-binding protein is also produced by *M. incognita*. *At3g22620* is another LTPg gene also strongly downregulated in syncytia according to Szakasis et al. [17] but belongs to group 3, while *LTPg5* belongs to group 1. *At3g22620* is also downregulated in roots infected by *Plasmodiophora brassicae* [20].

Our results show that the *LTPg5* gene is mainly expressed in roots. Besides that, there is some expression in stems, flower sepals, and older rosette leaves, especially after flowering or during senescence. An expression during leaf senescence has been reported before [21]. In addition, we found induction in rosette leaves after infection with plant pathogenic bacteria (*P. syringae*) and fungi (*B. cinerea*). The induction by *P. syringae* might be due to flagellin, as [22] reported induction after the treatment of Arabidopsis plants with flg22. This gene was also strongly induced in response to *Phytophthora-parasitica*-derived Nep1 (necrosis and ethylene-inducing peptide1) peptide [22]. Additionally, during a thrips infection in our growth chamber, we found that the *LTPg5*::GUS plants, which were also infected, showed GUS expression around the thrips feeding sites (Figure S2) but this finding would require further investigation. The regulation of *LTPg5* expression involves the MAP (Mitogen-activated protein) kinase substrate MKS1. Its expression was upregulated in both an *mpk4* mutant and a 35S-MKS1 overexpression line as compared to wild type, along with the induction of PR genes encoding PR1 and PR2 and the SA biosynthetic enzyme ICS1 (encoded by SID2) [23].

Our results are in line with data available at Genevestigator [24] (Figure S1). Furthermore, according to these data, the *LTPg5* gene is also induced by infection with mildew and *Sclerotinia sclerotiorum*. It was not induced by *Rhizoctonia solani* and viral infection. In contrast to this, the *At3g22620* gene is only induced by *P. syringae*, indicating that this gene might only be involved in resistance against bacteria such as *P. syringae*. This is supported by data from [19], showing that the overexpression of *At3g22620* resulted in enhanced resistance against *P. syringae* but not *H. schachtii*.

### 3.3. LTPg5 Is Involved in Plant Resistance against Plant Pathogenic Nematodes

The regulation of the *LTPg5* gene pointed to a function in plant defense, which we studied with overexpression lines and a T-DNA mutant. There were less female and male *H. schachtii* nematodes developing on both overexpression lines, while on the mutant the number of females was increased. Barnes et al. [19] also reported fewer female nematodes on 2 of 3 overexpression lines, while the overexpression of *At3g22620* had no effect on the number of female nematodes. We also measured the size of syncytia and of female nematodes. Only in one overexpression line there was a small but significant decrease in syncytium size, and the larger size in the mutant was not significant, while the size of female nematodes was smaller on both overexpression lines but larger on the mutant line. It seems that, even though the effect on syncytium size was only small, the LTPg5 protein affects syncytium development, which then also affects the development of the nematodes. In case of *M. incognita*, we measured the number of galls, which was significantly reduced in both overexpression lines, however, there was no effect of the mutation. Thus, the *LTPg5* gene is involved in resistance against both nematode species tested, *H. schachtii* and *M. incognita*.

### 3.4. The Role of LTPg5 in Plant Resistance against Different Pathogens

The induction of the gene in response to plant pathogenic bacteria (*P. syringae*) and fungi (*B. cinerea*) indicated that *LTPg5* might also be involved in resistance against pathogens other than nematodes. We also tested this with both overexpression lines and the mutant. Both overexpression lines supported less bacteria while the mutant showed the opposite effect. Barnes et al. [19] had also tested the resistance of overexpression lines for *LTPg5* and *At3g22620* against *P. syringae*. They reported that all lines supported a significantly lower number of bacteria than the wild type. Interestingly, the *At3g22620* overexpression lines, as mentioned before, were not more resistant to *H. schachtii*. Our *LTPg5* overexpression lines were, in addition, significantly more resistant against *B. cinerea*, while the mutant was more susceptible. Recently, it was reported that several LTPg genes, among which was *LTPg5*, were involved in resistance against a non-host powdery mildew (*Blumeria graminis* f. sp. *hordei*) in Arabidopsis [15]. The authors determined penetration frequency at 3 dpi, which was higher for a *LTPg5* mutant. However, no overexpression lines were tested. The gene expression data at Genevestigator (Figure S1) show that *LTPg5* is the only Arabidopsis LTPg gene (at least of those represented by the GeneChip) that is strongly induced by different fungi and *P. syringae*, followed by At1g62790, while *At3g22620* is only induced by *P. syringae*. Thus, *LTPg5* is probably the most important LTPg gene generally involved in resistance against pathogenic nematodes, bacteria, and fungi. As we found that the promoter::GUS construct was also induced by thrips infection, it might be interesting to test if the gene is also involved in resistance against insects.

The overexpression of LTP genes in plants has been carried out in a number of plant species, which has resulted in increased resistance against different pathogens (reviewed by [25]). For example, the overexpression of a barley LTP in transgenic tobacco enhanced the resistance of the plants towards *P. syringae* [26]. A wheat LTP was found to enhance biotic but also abiotic stress tolerance in Arabidopsis [27]. The expression of Ace-AMP1, a LTP from onion, in indica rice, lead to enhanced resistance against bacterial and fungal pathogens [28].

### 3.5. Possible Mode of Action of LTPg5 Related to Plant Resistance

Although it has been shown that a number of LTP genes are involved in plant resistance, their mode of action is poorly understood. For some LTPs, a direct antimicrobial activity has been demonstrated in vitro. The onion LTP Ace-AMP1, for instance, had strong antimicrobial activity in vitro against all fungal species tested and a few bacteria [5]. Transgenic rice plants expressing this LTP were more resistant against bacterial and fungal pathogens, probably due to the direct antimicrobial activity of the protein [28].

In case of the here described LTPg5 encoded by *At3g22600*, it is not clear if the function in plant resistance is due to a direct or indirect effect on the pathogens. The LTPg5 protein contains a typical LTP domain with eight cysteine residues and a basic pI (8.22), which is typical for antimicrobial peptides. However, there is a C-terminal extension with a binding site for the GPI anchor that would probably attach the whole protein to the plasma membrane with the LTP domain facing the apoplast. This extension has a pI of 4.11, giving the whole protein a pI of 4.8. One might speculate that the LTP domain could be cleaved off from the C-terminal extension resulting in an AMP with direct antimicrobial activity. This possible in vitro antimicrobial activity could be tested by expressing the LTP domain of LTPg5 for antimicrobial assays. The subcellular localization on the other hand could be analyzed with a GFP fusion or antibodies.

Another possible explanation how LTPg5 might exert a role in plant protection could be by signaling for expression of general resistance mechanisms. A prominent example for such a function is the Arabidopsis LTP DIR1, which is involved in signaling for systemic acquired resistance [8,29]. However, DIR1 does not have a GPI anchor. Our expression analysis of *PR* protein genes in *P. syringae* infected plants points to a signaling function of LTPg5. The expression of the salicylic acid inducible *PR1* gene, a marker for systemic resistance responses, was blocked in the mutant following infection with *P. syringae*. In contrast to this, in the case of *Pdf1.2a* and *PR4,* the expression was relaxed in the mutant while there was no induction after infection. *PR4* is regulated by ethylene, while *Pdf1.2a* is regulated by both ethylene and jasmonic acid. Thus, our results indicate that *LTPg5* is involved in the induction of salicylic acid responsive genes while inhibiting the expression of ethylene inducible genes in response to infection with *P. syringae*. Of course, these results will have to be validated by the use of mutants in the respective hormone pathways, for instance, by crossing them with the mutant and an overexpression line.

One possible mechanism by which LTPg5 might lead to the induction of defense-related genes could be by association with a receptor-like kinase. This has been shown for several Arabidopsis GPI proteins and has also been found to occur in mammals [30]. However, this has not been reported for LTPg proteins.

## 4. Materials and Methods

### 4.1. Bioinformatic Characterization of GPI-Anchored LTPg Genes and Proteins

The information on Arabidopsis LTPg genes regarding the nomenclature was adopted from [11], who reported 34 LTPg from Arabidopsis genome. The sequence data for gDNA, CDS, and the amino acid sequences of all LTPg genes were retrieved from TAIR (https://www.arabidopsis.org/). The characterization of LTPg genes and proteins was done based on different attributes, such as length of gDNA, CDS, length of proteins, the number and position of introns and exons, and exact localization of LTPg genes on their corresponding chromosome. The online SignalP 4.1 Server was used to predict the signal peptide cleavage site present in the amino acid sequences of LTPg proteins [31] (http://www.cbs.dtu.dk/services/SignalP/). ExPASy was used to determine isoelectric points [32] and is available at https://web.expasy.org/compute_pi/. The omega site corresponding to the GPI-anchor was predicted by using PredGPI (available at http://gpcr2.biocomp.unibo.it/gpipe/pred.htm) [33]. Likewise, to compute the grand average of hydropathy (GRAVY), the Sequence Manipulation Suite (SMS) [34], was accessed at http://www.bioinformatics.org/sms2/proteingravy.html.

The exact localization of LTPg genes on their corresponding chromosome was carried out by using MapChart [35] with default settings. Multiple sequence alignment was performed with the help of MEGA v. 7.0 by using ClustalW. Phylogenetic analysis of LTPg proteins was done by MEGA v. 7.0 [36]. For phylogenetic analysis, multiple sequence alignment was performed through ClustalW, and the tree was developed using maximum likelihood based on the Whelan and Goldman (WAG) model under default parameters. The use of the Whelan and Goldman (WAG) model was first calculated through substitution model determination. Moreover, to find conserved domains among LTPg proteins, we used the online MEME SUITE tool [37]. Gene structure analysis on the basis of the position, numbers, and length of untranslated regions (UTRs), exons, and introns was carried out using Gene Structure Display Server 2.0 (GSDS) [38] available at http://gsds.cbi.pku.edu.cn/.

### 4.2. Cloning of Binary Vectors

We used the vector pPZP3425 [39] for overexpression analysis. For cloning of the overexpression vector, the LTP coding sequence was amplified from genomic DNA by PCR using primers At3g22600forNcoI and At3g22600revBamHI (Table S3) containing the restrictions sites NcoI and BamHI. The PCR fragment was digested with NcoI and BamHI and ligated into the vector pPZP3425 digested with the same enzymes. The final construct was confirmed by sequencing.

A promoter::GUS fusion was cloned in the vector pMAA-Red [40]. The promoter region was amplified by PCR using Arabidopsis genomic DNA as a template. PCR primers pAt3g22600forEcoRI and pAt3g22600revNcoI (Table S3) included restriction sites for EcoRI and NcoI. After digestion, the PCR fragment was ligated to the large vector fragment of pMAA-Red digested with the same enzymes, thus replacing the 35S promoter by the LTP promoter. The construct was verified by sequencing.

### 4.3. Plant Material and Growth Conditions

Arabidopsis plants were cultivated on soil in a growth chamber at 25 °C in a 16 h light and an 8 h dark cycle for seed production. Arabidopsis seeds (ecotype Columbia) were surface sterilized for 20 min in 6 % (*w*/*v*) sodium hypochlorite and were subsequently washed three times with sterile water for in vitro growth on either Murashige and Skoog (MS) medium or Knop medium [41].

### 4.4. Screening of T-DNA Insertion Mutants

Seeds of the LTP knock-out mutant (SAIL_329_H03) were obtained from The European Arabidopsis Stock Centre (NASC) [42,43]. Genomic DNA from the leaves of 2-week-old seedlings was isolated using Edwards’s solution (200 mM Tris HCl pH 7.5, 250 mM NaCl, 25 mM EDTA, 0.5% SDS) and the lines with T-DNA insertions were screened by PCR (Figure 8) using the primers noted in Table S3.

### 4.5. Arabidopsis Transformation

Binary vectors were introduced into *Agrobacterium tumefaciens* GV3101 by a freeze–thaw method [44] for the transformation of *Arabidopsis* plants by a modified floral dip method [45]. For transformation with pPZP3425, seedlings were selected on MS medium with 50 μg/mL kanamycin and 250 μg/mL timentin at 22 °C with a photoperiod of 16 h light and 8 h dark until kanamycin-resistant seedlings could be clearly identified. Seedlings that were resistant to kanamycin were transferred to soil for seed production. For transformation with pMAA-Red, transformed seeds were identified as described by Ali et al. [40] and were also transferred to soil for seed production.

For each promoter::GUS construct, 12 independent transgenic plants were generated and tested for GUS activity to choose a representative line, which was grown further to produce homozygous seeds. For overexpression lines, 12 independent transgenic T2 lines were generated and applied to RT-PCR using the primers described in Table S3 to select the best expressing lines. These were then made homozygous for resistance tests.

### 4.6. Resistance Tests with H. schachtii and M. incognita

We used 0.2 concentrated Knop medium supplemented with 2% sucrose [41] for multiplying *H. schachtii* on mustard (*Sinapis alba* cv. Albatros) roots in vitro under sterile conditions. The cysts were collected from the mustard stock cultures and hatching of J2 larvae was stimulated by soaking the cysts in 3 mM ZnCl_2_. The larvae were washed three times in sterile water and were then resuspended in 0.5% (*w*/*v*) gelrite (Duchefa, Haarlem The Netherlands) for infection of Arabidopsis roots. The Arabidopsis seedlings were grown on Knop medium for 12 days. The roots were then inoculated with approximately 50–60 J2 larvae per plant. Three independent experiments were performed with 5 Petri dishes each. One Petri dish contained approximately 10 seedlings. Female and male nematodes were counted at 14 dpi and the number of males and females per cm of root length was calculated. At 14 dpi, female nematode and syncytia associated with female nematodes were photographed using an inverse microscope (Axiovert 200M; Zeiss, Hallerbergmoos, Germany) having an integrated camera (AxioCam MRc5; Zeiss) and were measured according to Ali et al. [46].

The egg masses of *M. incognita* were harvested from sterile stock cultures propagated on cucumber roots growing on B5 agar medium. Hatching of second-stage juveniles (J2) of *M. incognita* was stimulated by soaking the egg masses in sterile water by incubating for 2–4 days at room temperature in the dark. For sterilization, freshly hatched J2 were sterilized by incubation for 5–10 min in sterilization solution (0.002% HgCl_2_), followed by 3 to 4 washings with sterile distilled water. After sterilization, the suspension of J2 larvae was added with 0.7% gelrite and 12-day-old Arabidopsis plants growing on Knop medium were inoculated with these larvae. The number of galls was counted after 14 dpi.

### 4.7. P. syringae Infection Assay on Plates

The infection assay was done according to [18] using the strain *P. syringae* pv *tomato* DC3000. *P. syringae* was cultured on King’s B medium and the cells were suspended in 10 mM MgCl_2_ at a concentration of 5 × 10^5^ CFU/mL. Arabidopsis plants were grown on half strength MS medium with 0.3% phytagel at 24 °C with a 12 h light/ 12 h dark photoperiod for 2 weeks. They were inoculated by flooding with the suspension of *P. syringae* in 10 mM MgCl_2_ until the plants were completely submerged in inoculum. After flooding, the inoculum was removed from the plates and plates were put back to the growth chamber. One hour after inoculation, and for each investigated line, 4 seedlings (only aerial parts) were taken and surface sterilized with 5% H_2_O_2_ for 3 min and washed three times with sterile distilled water. This pooled sample of 4 seedlings was homogenized in 10 mL of sterile distilled water using a mortar and pestle, and diluted samples were plated onto King’s B medium containing rifampicin (50 μg/mL). Colonies were counted after 24 h using proper diluted samples. The counting of bacteria was repeated at 3 dpi and the CFU was normalized as CFU/mg using the total weight of inoculated seedlings. Bacterial numbers were evaluated in three independent experiments.

### 4.8. Resistance Test against B. cinerea

Arabidopsis plants were grown on soil under short day conditions. *B. cinerea* was grown on potato dextrose plates (38 g/liter potato dextrose agar). After two weeks, the spores of the fungus were harvested in sterile water and passed through sterile cotton cloth. A total of 10 μL of spores were put on the spore counter (Haemocytometer Thoma, area 0.0025 mm^2^, depth 0.1 mm, LO- Laboroptik, Lancing, UK) and counted under a microscope (Binocular Leitz Dialux 22 Leica Microsystems, Wetzlar, Germany). The spore suspension was adjusted to 10^5^ spores/mL using ½ strength potato dextrose broth. Drops of 5 μL spore suspension were deposited on the upper surface of 4-week-old plants on the left or right side for all the leaves to have uniformity in the visual observations. The plants were covered with plastic covering to maintain high humidity. For GUS staining, the leaves were stained at different time points. For assessing the resistance response of overexpression lines and mutants, the lesion diameter was determined with the help of a meter rod at 5 dpi and the photo of each leaf was taken with a camera (Digital Camera Nikon Coolpix S8200, Nikon, Japan). For each line, 3 plants were used and the experiment was performed in triplicate.

### 4.9. RNA Isolation

Samples from different plant parts were immediately frozen in liquid nitrogen. Syncytia were excised from infected roots at different time point (5 and 15 dpi). Total RNA was extracted using a NucleoSpin^®^ RNA Plant kit (genXpress, Wiener Neudorf, Austria) according to the manufacturer’s instructions, including DNase digestion. However, this DNase treatment did not completely digest the DNA present in the sample, so the DNA was therefore digested using Ambion^®^ DNA-free™ DNase Treatment and Removal Reagents (Invitrogen, Thermos Fisher Scientific, Vienna, Austria). The quality of the RNA was assessed using an Agilent 2100 Bioanalyzer (Agilent Technologies, Palo Alto, USA). RNA was quantified using NanoDrop (NanoDrop™ 2000c from PEQLAB). Isolated RNA was stored immediately at −80 °C.

### 4.10. Reverse Transcriptase (RT-PCR) and Quantitative Real Time PCR (qRT-PCR)

Semi quantitative RT-PCR was done using the RT-PCR Master Mix (USB) following the manufacturer’s instructions. For cDNA synthesis Superscript III reverse transcriptase (Invitrogen) and random primers (dN_6_), according to the manufacturer’s instructions, were used. The qRT-PCR was performed on an ABI PRISM 7300 Sequence Detector (Applied BioSystems, Thermos Fisher Scientific, Vienna, Austria). Each qRT-PCR sample contained 12.5 μL Platinum SYBR Green qPCR SuperMix (Thermos Fisher Scientific, Vienna, Austria), 2 mM MgCl_2_, 0.5 μL forward and reverse primer (10 μM) each, 2 μL cDNA, and water to make a total reaction volume of 25 μL. The RT-PCR and qRT-PCR forward and reverse primer pairs used for different genes are given in Table S3. Primer efficiencies were determined by using different concentrations of cDNA from seedlings of wild type. Control reactions with no cDNA template ruled out false positives. Dissociation runs were performed to make sure that there was no formation of primer dimers. The 18S gene was used as an internal reference in all qRT-PCR reactions. The results were calculated using the Sequence Detection Software SDS v2.0 (Applied BioSystems, Thermos Fisher Scientific, Vienna, Austria). The relative expression was calculated by the (1+E)^−ΔΔCt^ method [47].

### 4.11. GUS Reporter Analysis

The histochemical detection of GUS activity was performed according to [48]. GUS staining of different plant parts was performed by the incubation of the plant tissues in X-gluc (Biomol, Hamburg, Germany) in 0.1 M sodium phosphate buffer pH 7.0, 0.1% Triton-X 100, 0.5 mM K_3_[Fe(CN)_6_], 0.5 mM K_4_[Fe(CN)_6_] and 10 mM Na_2_EDTA at 37 °C for different time points at different stages of plants. After staining, chlorophyll was removed from photosynthetic tissues with 70% (*v*/*v*) ethanol. The seedlings and different plant parts were stained before and after flowering. Similarly, for GUS staining of syncytia and galls, the infected roots of promoter::GUS plants were incubated with X-gluc at 37 °C. The staining in syncytia, galls and uninfected roots was performed at different time points and photographed under an inverse microscope (Axiovert 200M; Zeiss, Hallerbergmoos, Germany) having an integrated camera (AxioCam MRc5; Zeiss).

### 4.12. Statistical Analysis

Data regarding the number of nematodes, the number of galls, the size of female nematodes and syncytia, the number of CFU, and lesion diameter were analysed using single factor ANOVA (*p* < 0.05), while the mean comparisons were performed using a least significant difference (LSD) test at a 95% level of confidence.

## Supplementary Materials

Supplementary materials can be found at https://www.mdpi.com/1422-0067/21/5/1774/s1.
