# Supplementary material for: The Arabidopsis GPI-Anchored LTPg5 Encoded by At3g22600 Has a Role in Resistance against a Diverse Range of Pathogens
Source: Int J Mol Sci. 2020 Mar 5;21(5):1774. doi: 10.3390/ijms21051774 (PMC7084707; doi:10.3390/ijms21051774)
Supplement: Supplementary file 1 [file ijms-21-01774-s001.pdf]

Supplementary materials

Table S1. Different attributes of *LTPg* genes and proteins.

Accession number	Chr #	*LTPg*	Protein length (AA)	Protein mol. wt.	(pI)	Start and end point of gene on chromosome	Predicted ω-site	gDNA length	CDS length	SP cleav. site	GRAVY
AT1G03103	1	19	171	25316.7	4.55	747197-748057	147	861	516	23-24	0.261
AT1G05450	1	21	205	20621.1	6.98	1599911-1601214	179	1304	618	27-28	0.199
AT1G18280	1	3	180	18072.5	7.92	6294333-6295364	158	1032	543	22-23	0.209
AT1G27950	1	1	193	19758.7	7.73	9740704-9742159	165	1456	582	22-23	0.226
AT1G36150	1	23	256	25906.1	7.72	13528257-13529470	225	1214	771	21-22	0.154
AT1G55260	1	6	227	25307.6	6.7	20614633-20616435	203	1803	684	NO	-0.267
AT1G62790	1	7	150	15711.3	7.96	23252313-23253743	125	1431	453	25-26	0.308
AT1G70250	1	32	799	87507.7	6.42	26452965-26456088	774	3124	2400	19-20	-0.1
AT1G73550	1	8	152	16509.3	8.41	27647756-27648536	124	781	459	23-24	0.384
AT1G73560	1	9	147	15512	7.63	27649695-27650471	125	777	444	21-22	0.406
AT1G73890	1	10	193	9880.7	7.96	27787903-27788658	168	756	582	24-25	0.448
AT2G13820	2	11	169	6816.2	6.98	5774146-5776289	146	2144	510	23-24	0.62
AT2G27130	2	12	176	18088.6	4.44	11595292-11596542	149	1251	531	20-21	0.252
AT2G44290	2	13	205	21578.7	6.49	18305201-18306251	177	1051	618	23-24	0.155
AT2G44300	2	14	204	21705.8	6.88	18307309-18308349	180	1041	615	25-26	0.088
AT2G48130	2	15	183	18852.4	8.66	19685066-19685993	158	928	552	24-25	0.134
AT2G48140	2	16	200	20144.3	7.96	19686409-19687664	176	1256	603	20-21	0.364
AT3G22570	3	17	116	12208	4.28	8000515-8001238	96	724	351	24-25	0.276
AT3G22580	3	18	127	14021.1	4.71	8002762-8003145	105	384	384	26-27	0.199
AT3G22600	3	5	170	17305.4	4.8	8006508-8007471	146	964	513	24-25	0.041
AT3G22620	3	20	203	20778.6	6.25	8008534-8009590	172	1057	612	21-22	0.151
AT3G43720	3	2	193	19175	4.3	15615386-15617149	165	1764	582	22-23	0.544
AT3G58550	3	22	177	19291.2	8.49	21649364-21650287	152	924	534	29-30	-0.044
AT4G08670	4	4	208	20851.5	7.15	5536699-5538210	179	1512	627	25-26	0.204
AT4G12360	4	24	161	16329.9	5.72	7327992-7329192	138	1201	486	23-24	0.563
AT4G14805	4	25	219	23546.6	4.05	8502374-8503199	192	826	660	22-23	0.188
AT4G14815	4	26	156	16773.2	8.33	8505043-8505952	128	910	471	22-23	0.184
AT4G22630	4	27	162	17729.4	7.61	11909368-11909945	137	578	49	29-30	0.065
AT4G22640	4	33	116	12504.4	4.52	11911600-11912107	93	508	351	27-28	0.359
AT4G22650	4	34	156	17405	7.78	11913557-11914027	128	471	471	22-23	0.185
AT4G22666	4	28	160	16586.7	4.47	11917057-11917879	135	823	483	22-23	0.05
AT5G09370	5	29	158	16163.7	8.2	2909363-2910545	134	1183	447	24-25	0.556
AT5G13900	5	30	151	16532	8.5	4481232-4481889	120	658	456	22-23	0.073
AT5G64080	5	31	182	17971.4	6.98	25645263-25646735	157	1473	594	28-29	0.637

LTPgs are numbered according to Edstam et al. (2013)

Table S2. The transcriptome data of GPI-anchor coding LTP genes in syncytia vs. roots (Szakasits et al., 2009).

Gene	*LTPg*	Syncytia	Root	Syncytia vs. root
LTPg genes significantly upregulated in syncytia				
At5g64080	31	7.5	2.6	4.9
At2g27130	12	6	3.8	2.2
LTPg genes significantly downregulated in syncytia				
At3g22600	5	4.2	12.7	-8.5
At2g48140	16	3.2	9.5	-6.3
At2g48130	15	2.9	9.1	-6.2
At5g13900	30	2.7	8.5	-5.8
At3g22620	20	3.7	9	-5.3
At3g22570	17	4.2	7.8	-3.6
At4g22640	33	3.9	5.8	-1.9
At3g58550	22	4	5.5	-1.5
At2g13820	11	6.3	7.5	-1.2
At1g05450	21	2.7	3.4	-0.7
LTPg genes non-significantly regulated in syncytia				
At4g14815	26	3.2	4	-0.8
At3g43720	2	3.3	3.5	-0.2
At4g12360	24	2.8	3.2	-0.4
At4g22650	34	2.5	2.7	-0.2
At1g18280	3	3.1	2.7	0.4
At2g44300	14	7.2	7.7	-0.5
At1g62790	7	9.9	9.6	0.3
At1g55260	6	2.7	2.7	0
At1g36150	23	3.8	4.1	-0.3
At4g08670	4	2.7	2.9	-0.2
At5g09370	29	3	3.1	-0.1
At1g27950	1	2.6	2.9	-0.3
At1g70250	32	3.5	3.7	-0.2
At1g73560	9	2.2	2.2	0
LTPg genes not present on the GeneChip				
At4g14805 (*LTPg25*), *At4g22630* (*LTPg27*), *At2g44290* (*LTPg13*), *At1g03103* (*LTPg19*), *At1g73550* (*LTPg8*), *At3g22580* (*LTPg18*), *At1g73890* (*LTPg10*), *At4g22666* (*LTPg28*)				

LTPgs are numbered according to Edstam et al. (2013). Colours indicate in which group the LTPg is located (see Figure 2)

Table S3: Primers used in this work (restriction sites are underlined)

Name	Sequence	Amplification
At3g22600forNcoI	AAGAAACCATGGAAATGGAAATGG	Protein coding sequence of *At3g22600* from cDNA
At3g22600revBamHI	AAATGGATCCTCACTTGATTGCC	
At3g22600RTfor	CTCTACCTCTCCTAATCAGCAATG	semi-quantitative RT-PCR of *At3g22600*
At3g22600RTrev	GTTTTCGATCCATTACCTGGTC	
At3g22600qRTFor	CTCTACCTCTCCTAATCAGCAATG	qPCR of *At3g22600*
At3g22600qRTRev	GATCCATTACCTGGTCCTGAAGAG	
18Sfor	GGTGGTAACGGGTGACGGAGAAT	qPCR reference gene
18Srev	CGCCGACCGAAGGGACAAGCCGA	
pAt3g22600forEcoRI	GTTGAATTCTTTTGGAAGTTTAAAGCT	Promoter fragment of *At3g22600*
pAt3g22600revNcoI	TTCCATTTCCATGGTTTCTTGAAGATG	
SAIL_329_H03 LP	TAGACCGTCACAAAGACCACC	Mutant screening
SAIL_329_H03 RP	ACAACAAAGCAATATCACCCG	
LB3-Sail	TAGCATCTGAATTTCATAACCAATCTCGATACAC	T-DNA primer
Pdf1.2aRTfor	GCTAAGTTTGCTTCCATCATCACC	qPCR of *Pdf1.2a*
Pdf1.2aRTrev	GTGTGCTGGGAAGACATAGTTGC	
PR1RTfor	GTGACTTGTCTGGCGTCTCC	qPCR of *PR1*
PR1RTrev	ACGTGTGTATGCATGATCACATC	
PR4RTFor	CTGGACCGCCTTCTGCGGG	qPCR of *PR4*
PR4RTRev	AGCCTCCGTTGCTGCATTGGT	

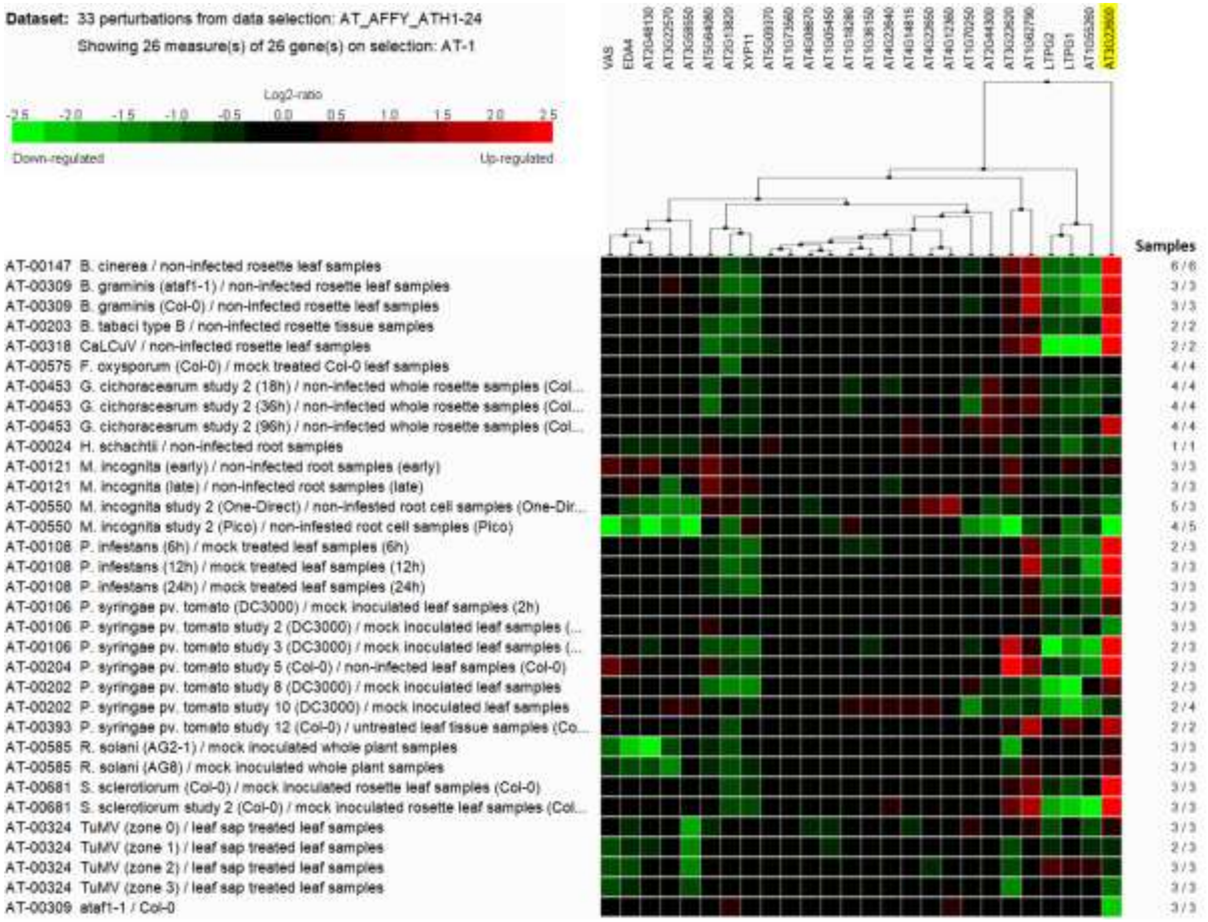

Figure S1: Expression of LTPg genes in response to biotic stress

Biotic interactions of 26 LTPg genes present on the Affymetrix gene chip were analyzed using Genevestigator [21]. VAS (*At5g13900 = LTPg30*), EDA4 (*At2g48140 = LTPg16*), XYP11 (*At2g27130 = LTPg12*).

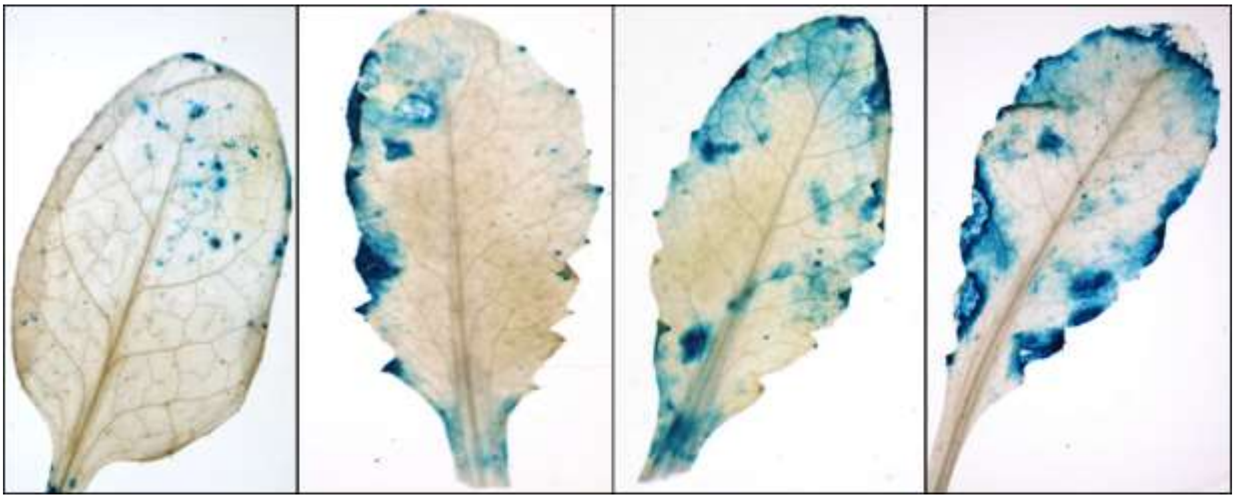

Figure S2: GUS staining due to the attack of thrips. 5 week old rosette leaves of a promoter::GUS line growing in a growth chamber on soil were stained for GUS activity after thrips infection. Left, leaf without thrips infection, other leaves with thrips infection.
